# Ossification du ligament vertébral commun postérieur lombaire et sciatalgie chez un patient tunisien

**DOI:** 10.11604/pamj.2014.18.114.4578

**Published:** 2014-06-04

**Authors:** Wassia Kessomtini, Wafa Chebbi

**Affiliations:** 1 Unité de Médecine Physique, CHU Taher Sfar Mahdia, 5100 Mahdia, Tunisie; 2 Service de Médecine Interne, CHU Taher Sfar Mahdia, 5100 Mahdia, Tunisie

**Keywords:** Sciatalgie, ossification du ligament vertébral commun postérieur, sciatica, ossification of the posterior longitudinal ligament

## Image en medicine

L'ossification du ligament vertébral commun postérieur (LVCP) est une pathologie hyperostosante relativement fréquente chez les sujets japonais, ce qui lui a valu la dénomination de « maladie japonaise ». Elle est caractérisée par une ossification hétérotopique au sein du LVCP pouvant aboutir à une compression médullaire et à une myélopathie sévère. Elle est le plus souvent de localisation cervicale et doit amener à chercher prioritairement une anomalie du métabolisme phosphocalcique, en particulier un rachitisme vitamino-résistant hypophosphatémique familial ou une hypoparathyroïdie, une hyperostose vertébrale engainante ou une chondrocalcinose articulaire. Nous rapportons un cas exceptionnel d'ossification du LVCP de localisation lombaire chez un patient tunisien. Il s'agissait d'un homme de 30 ans, sans antécédents pathologiques, adressé à l'unité de Médecine physique pour une lombosciatique S1 gauche tronquée évoluant depuis 3 mois et gênant la station assise et les activités de la vie quotidienne. Il n'y avait pas de trouble vésico-sphinctérien, ni de limitation de périmètre de marche. L'examen montrait un syndrome rachidien avec un signe de Lasègue positif à gauche. L'examen neurologique et des articulations périphériques était normal. Les radiographies standard du rachis lombaire montraient une ossification du LVCP lombaire à l’étage L5 et S1. Le scanner du rachis lombaire confirmait la calcification du LVCP en L5 et S1 avec un effet de masse sur le contenu canalaire essentiellement en postéro-para-médian gauche. Le bilan étiologique, phosphocalcique, était négatif. Une laminectomie de décompression du rachis lombaire et une excision de l'ossification étaient réalisée suivi d'une rééducation adaptée. L’évolution était favorable avec disparition complète de la symptomatologie.

**Figure 1:**
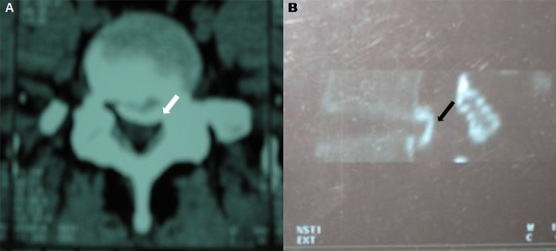
TDM du rachis lombaire: A) en coupe axiale: ossification du ligament vertébral commun postérieur en L5 et S1 (flèche blanche); B) en reconstruction dans le plan sagittal: hyper-densité du ligament vertébral commun postérieur à l’étage L5 et S1 (flèche noire)

